# Temporal Variability of the Dominant Fecal Microbiota in Healthy Adult Cats

**DOI:** 10.3390/vetsci11010031

**Published:** 2024-01-13

**Authors:** Chi-Hsuan Sung, Sina Marsilio, Rachel Pilla, Yu-An Wu, Joao Pedro Cavasin, Min-Pyo Hong, Jan S. Suchodolski

**Affiliations:** 1 Gastrointestinal Laboratory, Department of Small Animal Clinical Sciences, Texas A&M University, College Station, TX 77843, USA; csung@cvm.tamu.edu (C.-H.S.);; 2 UC Davis School of Veterinary Medicine, Department of Veterinary Medicine and Epidemiology, University of California, Davis, CA 95616, USA

**Keywords:** microbiota, dysbiosis index, variability, stability, bile acid, feline, *Clostridium hiranonis*, *Peptacetobacter hiranonis*

## Abstract

**Simple Summary:**

Alterations in the composition and function of the intestinal microbiota are linked to chronic enteropathy; however, the temporal variability of the intestinal microbiota is under-explored. This study aimed to evaluate the temporal variability of the feline dysbiosis index and the core bacterial taxa in healthy adult cats. A total of 142 fecal samples collected from 17 adult pet cats were included. The qPCR-based feline dysbiosis index was used to assess the fecal microbiota. The results showed temporal stability in the feline dysbiosis index in all healthy adult cats throughout the study. The feline dysbiosis index was consistently within the reference interval over two months in individual cats, and most targeted bacteria remained within their respective reference intervals. While individual variation was observed, the magnitude of impact was minimal compared to disease status and antibiotic use. In conclusion, our findings show the temporal stability of the feline dysbiosis index in healthy adult cats in the absence of perturbations.

**Abstract:**

While shifts in gut microbiota have been studied in diseased states, the temporal variability of the microbiome in cats has not been widely studied. This study investigated the temporal variability of the feline dysbiosis index (DI) and the abundance of core bacterial groups in healthy adult cats. The secondary aim was to evaluate the relationship between the fecal abundance of *Clostridium hiranonis* and the fecal concentrations of unconjugated bile acids. A total of 142 fecal samples collected from 17 healthy cats were prospectively included: nine cats with weekly collection over 3 weeks (at least four time points), five cats with monthly collection over 2 months (three time points), and three cats with additional collections for up to 10 months. The DI remained stable within the reference intervals over two months for all cats (Friedman test, *p* > 0.2), and 100% of the DI values (*n* = 142) collected throughout the study period remained within the RI. While some temporal individual variation was observed for individual taxa, the magnitude was minimal compared to cats with chronic enteropathy and antibiotic exposure. Additionally, the abundance of *Clostridium hiranonis* was significantly correlated with the percentage of fecal primary bile acids, supporting its role as a bile acid converter in cats.

## 1. Introduction

The gut microbiota plays a crucial role in maintaining feline health [1]. Studies on the gut microbiome have particularly focused on the changes between healthy and diseased populations [2]. Alterations in intestinal, fecal, or urinary bladder microbiota have been reported in cats with chronic enteropathies [3,4,5] and chronic kidney disease [6] and cats receiving antibiotics [7]. Evaluation of the microbiota as a diagnostic tool, treatment target, and disease prevention strategy requires a comprehensive understanding of its dynamics and stability over time.

Studies on the dynamics and stability of the feline microbiota over time have not been widely explored. On the other hand, in human medicine, the composition and function of the microbiota in healthy humans have proven stable over time without major perturbations in young adults and the elderly [8,9,10]. Even with perturbations, studies have also shown that the microbiota is resilient against long-term dietary intervention [11], short-course antibiotics [12,13,14], and environmental shifts [13] in healthy adults. Similarly, the fecal microbiota assessed by 16S rRNA gene sequencing remained stable over two months in six healthy research colony cats receiving 60 days of lactose as a placebo [15] and the major bacterial groups quantified by quantitative PCR did not change in 12 healthy pet cats receiving synbiotics over 6 weeks [16]. Conversely, antibiotics can induce temporary or long-term shifts in the microbial composition of individual cats [7,17], underlining the need to comprehend the microbiota’s stability and resilience in cats.

In addition to the structure of fecal microbiota, the metabolome—representing the functional aspect of the microbiota—is equally crucial [18,19,20]. Microbially derived metabolites are tightly related to the host’s health [20]. For example, intestinal microbes are involved in the deconjugation [21] and dehydroxylation [22] of bile acids (BAs) produced by the host. These microbial-derived BAs, especially secondary BAs, have anti-inflammatory [23,24] and anti-microbial effects [25]; therefore, they can modulate the immune response [26]. The 7α-dehydroxylation-mediated BA biotransformation is mostly limited to *Clostridium* spp. [22,27,28]. In dogs, *Clostridium hiranonis* (also known as *Peptacetobacter hiranonis*) has been extensively studied and identified as the major convertor of primary BAs into secondary BAs [29]. A reduction in *C. hiranonis* in feces has been reported in dogs [30] and cats with chronic enteropathy (CE) [31]. Previous studies also showed that fecal primary BAs increased in cats with CE [31] and kittens receiving antibiotics [32]. The balance and composition of BAs have been linked to the composition of the microbiota in humans [33,34], rats [35], and dogs [36,37]. Therefore, exploring the interplay between the gut microbiota and BA composition is another approach to studying temporal stability. Understanding the temporal variability of fecal microbiota in cats is important for research and clinical purposes. While many studies have used untargeted sequencing methods to evaluate the gut microbiota, the inevitable batch effect and low reproducibility limit its clinical utility [38,39]. The absence of reference intervals for each bacterium complicates linking sequencing results to clinical aspects. However, despite these challenges, the comprehensive overview from sequencing provides robust insights for marker discovery.

A reproducible targeted qPCR-based feline dysbiosis index (DI) has been recently reported, and reference intervals (RIs) have been established [40]. The feline DI applied qPCR to quantify seven core bacterial groups that are prevalent in all healthy cats: *Bacteroides*, *Bifidobacterium*, *C. hiranonis*, *E. coli*, *Faecalibacterium*, *Streptococcus*, and *Turicibacter*. The feline DI is a mathematical modeling based on the quantification of these bacterial groups and allows for distinguishing the gut microbiota of cats with CE from healthy ones. The clinical application of feline DI requires a better understanding of the temporal variability of these bacterial taxa.

Therefore, the primary aim was to determine the short-term temporal variability of the feline DI and the targeted bacterial taxa in healthy adult cats. Additionally, to evaluate the magnitude of the impact of individual variation in healthy cats, samples from cats receiving antibiotics and cats with CE were also included. The secondary objective was to evaluate the association between *C. hiranonis* and BA metabolism in cats, as another measure to understand the normal function and stability of the feline fecal microbiota.

## 2. Materials and Methods

### 2.1. Animal and Sample Collection

This study was a single-center, prospective, observational longitudinal study. Seventeen clinically healthy cats between the ages of 2 and 13 years old (median: 5 years) were enrolled. All cats were considered clinically healthy based on the owner’s report and 8 out of 17 cats had blood work evaluated within one month before sample collection. Besides neuter or spay surgery, the cats had no history of disease or any gastrointestinal symptoms. None had received antibiotics for at least one year before sampling. All cats were primarily fed a commercial maintenance kibble diet supplemented with additional commercial canned food. They were exclusively indoor cats with no access to the outdoors. There were no significant disturbances in their living conditions or diet with no medications administered throughout the sampling period. All cats were staff-owned at the Texas A&M Small Animal Teaching Hospital, College Station, TX, USA.

We used the feline DI to characterize the major bacterial groups in 142 fecal samples obtained from all 17 cats. Two cats were from the same household and the owners were instructed to collect samples immediately when they watched the defecation of individual cats. Naturally passed fecal samples were collected weekly in ten cats (on days 0, 7, 14, and 21) and monthly in four cats (on days 0, 29, and 59). Among the ten cats with four time-point collections, three cats had additional collections, totaling 11, 21, and 34 times, respectively, for up to two months. For a more extended assessment of temporal variability, one cat had daily collection for a week spaced 1 month apart with a total of 17 samples, another cat had 11 collections over 10 months, and a third cat had 8 collections over 9 months. The detailed collection time points of each cat are provided in the Supplementary Data Excel Sheet-Collection Time Point. The owners were instructed to keep the fecal samples frozen at −20 °C in a regular family-use refrigerator before submission. Upon the arrival of the samples in the laboratory, the feline DI was analyzed within two days.

To better compare the magnitude of the inter-individual variations over time in the healthy cats and that associated with antibiotics or disease, we included 25 samples from eight cats (age range: 3–13 years, medium: 5.5 years) that received antibiotics (two with a single 8 mg/kg subcutaneous cefovecin injection and six with 15 mg/kg q12h oral amoxicillin–clavulanate for 7 days) and a single-time point collection from 68 cats with chronic enteropathy, obtained from a previous cross-sectional study [40].

The study was approved by the Texas A&M University Institutional Animal Care and Use Committee (IACUC 2021-0035 CA).

### 2.2. Quantitative PCR Analysis and Feline Dysbiosis Index

The protocols for the qPCR assays and feline DI were previously described [40]. Fecal abundances of various bacterial taxa, consisting of total bacteria, *Bacteroides*, *Bifidobacterium*, *Clostridium hiranonis*, *Escherichia coli*, *Faecalibacterium*, *Streptococcus*, and *Turicibacter* were quantified by validated qPCR assays. Briefly, DNA from fecal samples was extracted using a DNeasy PowerSoil Pro kit (QIAGEN, Germanton, MD, USA) following the manufacturer’s instructions. A mixture of 2 µL normalized DNA extract (5 ng/µL), 5 µL of SsoFast EvaGreen supermix (Bio-Rad Laboratories Inc., Hercules, CA, USA), 0.4 µL of each forward and reverse primer (400 nM), and 2.2 µL of DNA-free water was used for qPCR assays using a Bio-Rad C1000 Touch Thermal Cycler (Bio-Rad Laboratories Inc., Hercules, CA, USA). The protocol of the thermal cycle was as follows: initial denaturation at 98 °C for 2 min and then 40 cycles with denaturation at 98 °C for 3 s and annealing for 3 s. The annealing temperature and the primer set for each target are provided in the Supplementary Data Excel Sheet-qPCR Condition. All samples were analyzed in duplicate and the average of the two results was used for further analysis. Quantitative PCR results of the targeted bacterial groups were used to calculate a feline DI.

We further classified the feline DI into four groups. A DI < 0 and with all targeted taxa within the RI was considered normal. A DI < 0 but with any of the targeted taxa outside the RI was defined as minor shift in the microbiota. A DI between zero and one was defined as mild-to-moderate shift. A DI > 1 was classified as significant dysbiosis.

### 2.3. Fecal Concentrations of Unconjugated Bile Acids

To examine the association between *C. hiranonis* and BA metabolism, concentrations of unconjugated BAs (cholic acid, chenodeoxycholic acid, deoxycholic acid, lithocholic acid, and ursodeoxycholic acid) in the fecal samples were quantified using a validated gas chromatography coupled with mass spectrometry (GC-MS) method [31]. Leftover fecal samples from a total of 78 fecal samples from healthy cats and 22 samples from cats receiving antibiotics were included in the analysis. The collection time and the availability of each sample are documented in the Supplementary Data Excel Sheet-Collection Time Point.

### 2.4. Statistical Analysis

The dysbiosis index and log DNA of each targeted bacterial group from cats with samples collected at similar time points were compared. The results from the 9/17 cats that had samples collected at days 0, 7, 14, and 21 were used to evaluate the temporal variability of the feline DI and log DNA of each targeted bacterial group using Friedman tests. The results of the 4/17 cats with sample collected at days 0, 29, and 59 over 2 months were analyzed using Friedman tests as well. The remaining cats with individual time-points are descriptively presented in a table and figures. To gain a comprehensive understanding of the overall variability based on qPCR results, principal component analysis (PCA) plots were generated via MetaboAnalyst 5.0 (a free web-based platform). To evaluate the magnitude of impact of individual variation, the qPCR results from the samples of eight cats receiving antibiotics and a cohort of 68 cats with CE were included for the visualization of their distribution on a PCA plot. The correlation between the percentage of primary BAs (absolute amount of the sum of cholic acid and chenodeoxycholic acid/absolute amount of the total measured BAs) and fecal abundance of *C. hiranonis* was evaluated with Spearman correlation. Unless otherwise specified, the majority of statistical analyses were performed in GraphPad Prism 9.0 (GraphPad Software Inc., San Diego, CA, USA).

## 3. Results

### 3.1. Feline Dysbiosis Index and the Abundances of Targeted Bacterial Groups

**Figure 1:**
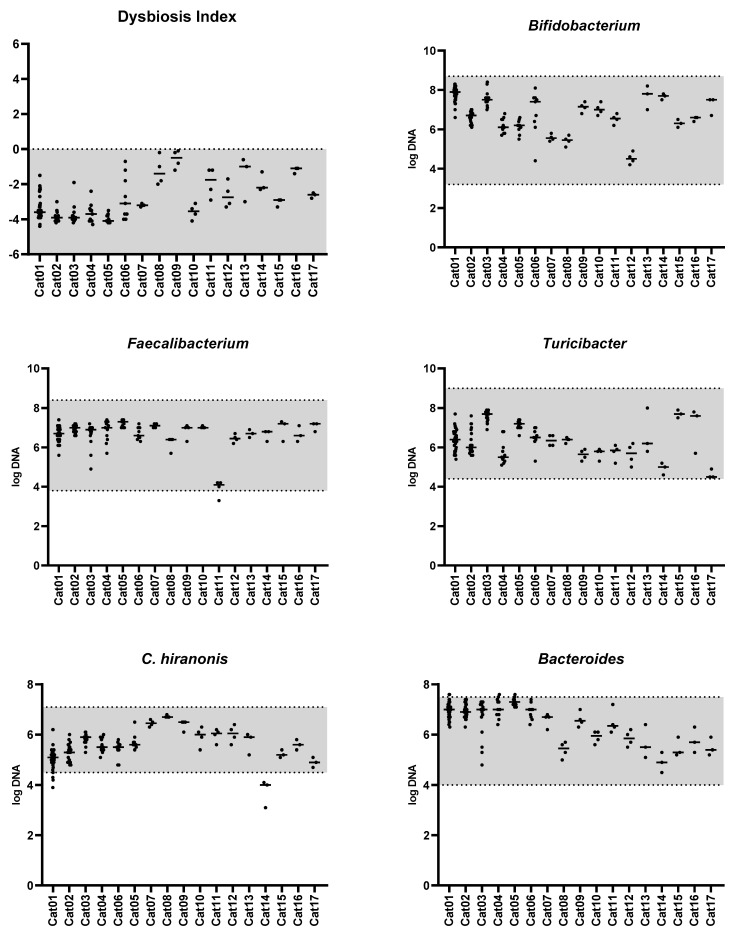

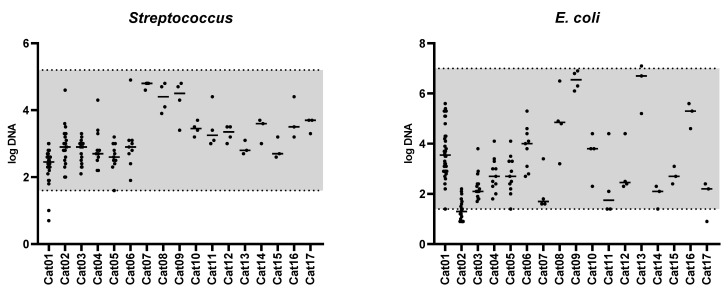
Feline dysbiosis index and the fecal abundances (log DNA/g feces) of the targeted bacterial groups in 17 healthy adult pet cats. The gray area represents the reference interval. The horizontal lines represent the median.

Figure 1 shows that the feline dysbiosis index (DI) was consistently within the reference interval (RI) (<0) across 142 samples from 17 healthy adult cats. The median DI was −3.5 (range: −0.1 to −4.4).

**Figure 2:**
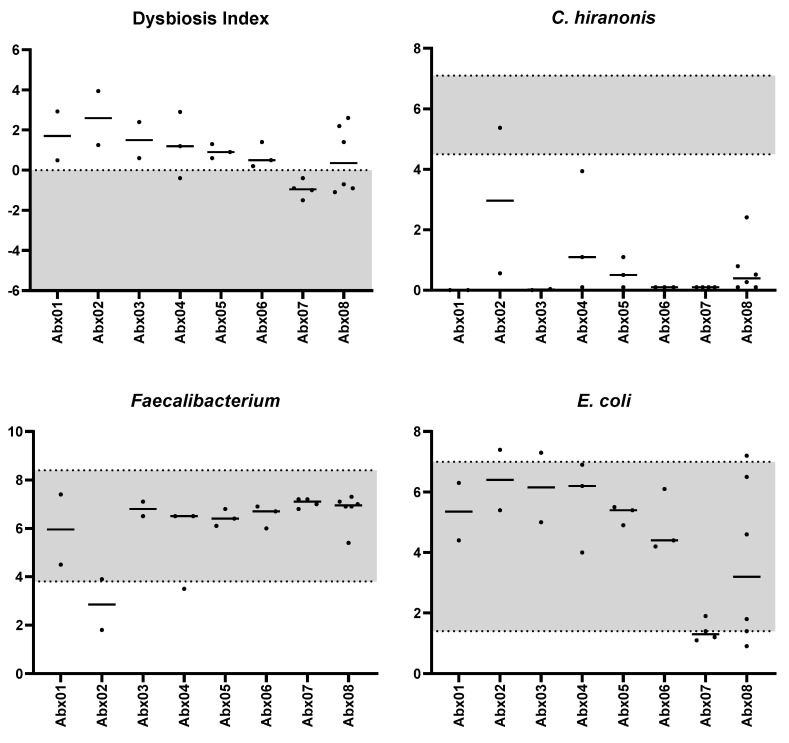
Feline dysbiosis index and the abundances (log DNA/g feces) of the selective targeted bacterial groups in eight cats exposed to antibiotics. The gray area represents the reference interval. The horizontal lines represent the median.

Notably, the abundance of targeted bacterial taxa within individual samples remained within their respective RIs most of the time (Figure 1). The median coefficients of variation were below 6.2% for *Bacteroides*, *Bifidobacterium*, *C. hiranonis*, *Faecalibacterium*, and *Turicibacter*, except *Streptococcus* and *E. coli* (Table 1). The abundances of *Bifidobacterium*,* Turicibacter*, *Faecalibacterium*, *C. hiranonis,* and *Bacteroides* were consistently within the RI in 100%, 100%, 99%, 96%, and 95% of the 142 samples, respectively. Because increased abundance of *Streptococcus* and *E. coli* is linked to dysbiosis [40], only the upper reference limit was considered for these two taxa. The abundances of *Streptococcus* and *E. coli* was above the upper limit of the RI in 0% and 1% of the 142 samples, respectively. Importantly, all of these samples were only slightly outside the RIs for all healthy cats. Conversely, 96% of samples from cats exposed to antibiotics showed a very low abundance of *C. hiranonis* (a median of 0.1 log DNA; range: 0.1–4.0, Figure 2).

In 142 samples from healthy cats, 94% were classified as normal DI, with 6% showing minor shifts (DI < 0 but at least one bacterial taxon outside the reference interval). Among the 25 samples from the eight cats receiving antibiotics, 11 (44%) were classified as significant dysbiosis, 6 (24%) showed mild-to-moderate changes, and 8 (32%) showed minor changes due to the low abundance of *C. hiranonis*, but the DI was <0 (Figure 2). In the 68 samples from cats with chronic enteropathy, 54% had significant dysbiosis, while 22%, 13%, and 10% showed mild-to-moderate shifts, minor shifts, and normal microbiota, respectively.

**Figure 3:**
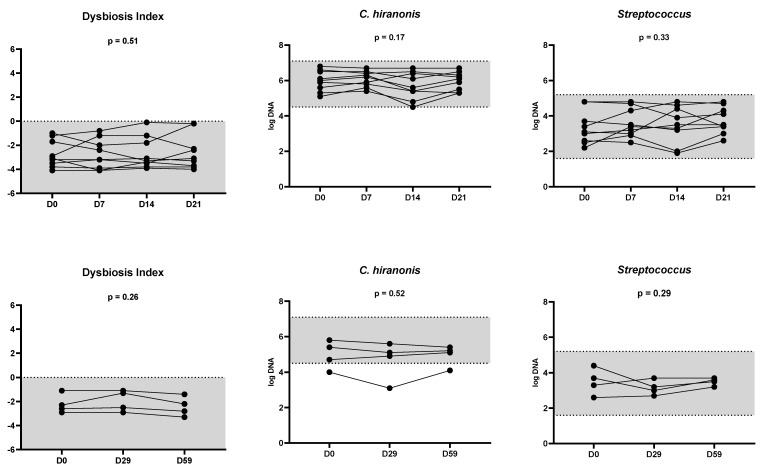
The feline dysbiosis index and representative fecal abundances of *C. hiranonis* and *Streptococcus* in nine cats with four-time points (days 0, 7, 14, and 21; the upper row) and four cats with three-time points (days 0, 29, 59; the lower row). The feline dysbiosis index and abundances of *C. hiranonis* and *Streptococcus* did not differ (all *p*-values > 0.1, Friedman tests) over two months. Only one cat had fecal abundance of *C. hiranonis* slightly lower than the reference interval, yet bile acid conversion persisted. Additionally, the DI of all cats was within the reference intervals over time. Gray areas represent the respective reference range.

**Figure 4:**
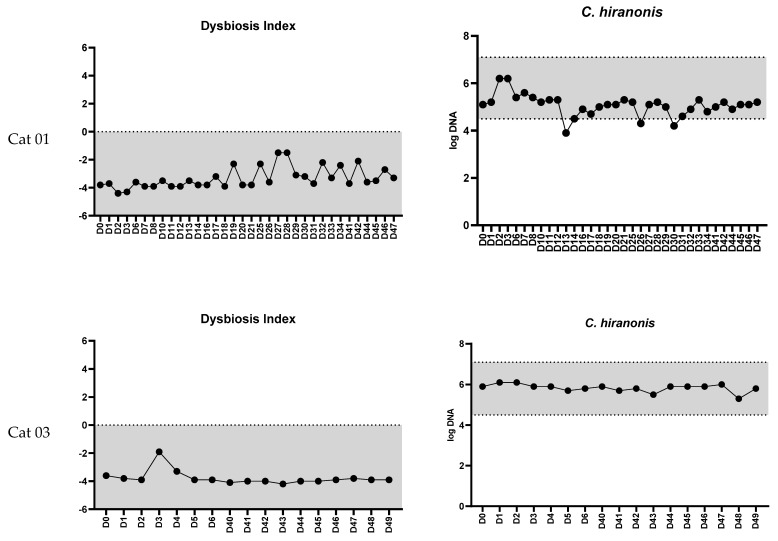

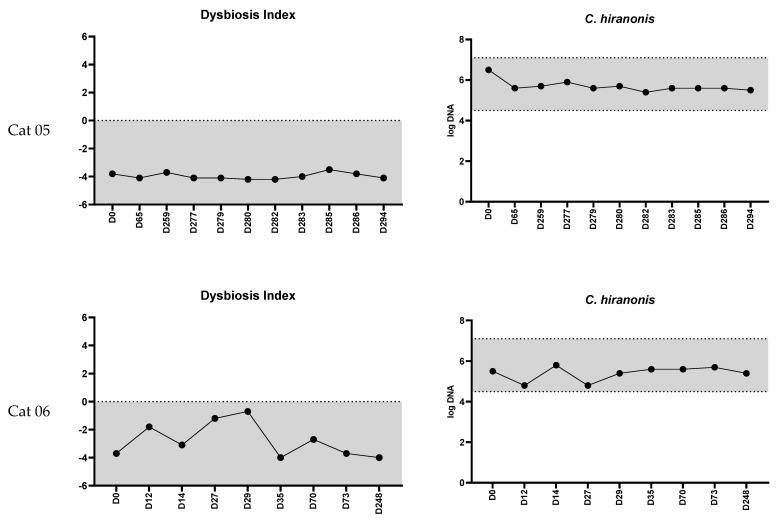
Examples of the feline dysbiosis index and the fecal abundances (log DNA/g feces) of *C. hiranonis* in four healthy adult pet cats across different time points (Cat 01: 47 days, Cat 03: 49 days, Cat 05: 294, and Cat 06: 248 days). For Cat 01, the abundance of *C. hiranonis* infrequently fell slightly below the reference interval (3 out of 34 samples). Nevertheless, the feline dysbiosis index remained within the normal range over an extended period in all cats.

In nine cats with samples collected weekly (days 0, 7, 14, and 21), the DI (*p* = 0.51) and fecal abundances of all targeted bacterial groups (all *p*-values > 0.1) did not differ over three weeks (Figure 3). Similarly, in four cats with samples collected monthly (days 0, 29, and 59), the DI and fecal abundances of all targeted bacterial groups did not differ (all *p*-values > 0.1) over two months. Figure 4 presents the DI and the abundance of *C. hiranonis* in healthy cats with additional collection time points up to 248 days. The DI consistently remained within the normal range (<0).

**Figure 5:**
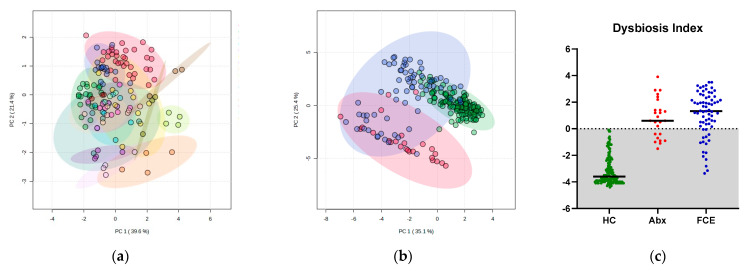
(**a**) PCA plot based on the qPCR results in 17 healthy cats across multiple time points. The samples are color-coded based on each cat. Each cat’s microbiota at a given time point had higher similarity to its own at other time points. (**b**) The PCA plots based on the qPCR results in healthy cats (green, consisting of all data points from Figure 5a), 25 samples from eight cats exposed to antibiotics (red), and a cohort of 68 cats with CE from a previous cross-sectional study (blue) [40]. The ellipse represents the 95% confidence regions. Regardless of the collection time, 142 samples from healthy cats cluster tightly, reflecting high homogeneity among the healthy cats. (**c**) Scatter plot of the dysbiosis index of 142 samples from 17 healthy cats (HC), 25 samples from 8 cats that received antibiotics (Abx), and 68 cats with chronic enteropathy (FCE). The dysbiosis index of healthy cats remained within the reference interval (gray area), while 76% of cats with FCE had a dysbiosis index above 0, including 54% with significant dysbiosis, 22% with mild-to-moderate shift, and 13% with minor shifts.

Based on the visualization of the PCA plot (Figure 5), samples from the same cat were clustered together, delineated by variable areas within the 95% confidence regions (Figure 5a). When consolidating all samples from healthy cats into one group, along with samples from eight cats subjected to antibiotics and those with feline chronic enteropathy, a small magnitude of impact of variability between healthy individuals was evident (Figure 5b). Healthy cats showed a tight clustering away from the other two groups. Additionally, cats with chronic enteropathy showed a larger 95% confidence region. While 68% of samples from cats receiving antibiotics had a DI below 0 (Figure 5c), 96% of these samples also had very low abundance of *C. hiranonis* (Figure 2). Moreover, these samples were distinct from samples of healthy cats based on a clear separation on the PCA plot (Figure 5b).

### 3.2. Relationship between the Fecal Bile Acid Profile and the Abundance of Clostridium hiranonis

**Figure 6:**
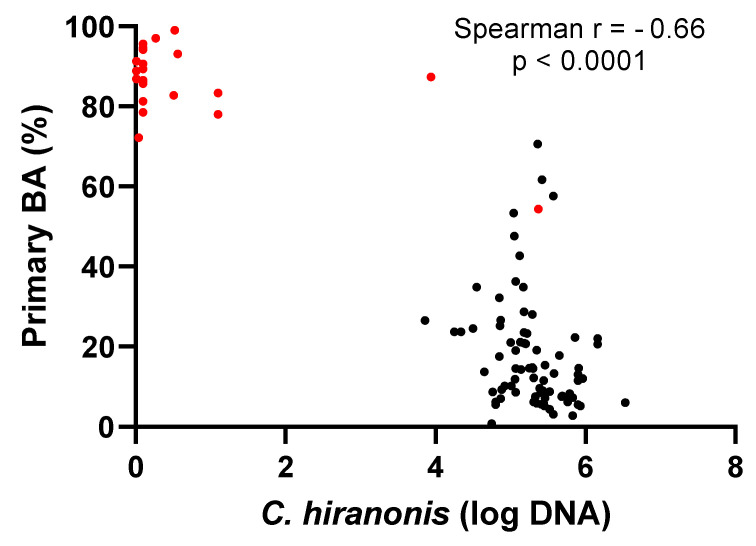
Scatter plot of *C. hiranonis* (log DNA) abundance and percentage of primary unconjugated bile acids. Primary bile acids with a median of 87% were dominant in 22 samples from cats receiving antibiotics (red dots), while healthy cats (black dots) had a median of primary bile acids of 14%, indicating the role of *C. hiranonis* in converting primary bile acids into secondary bile acids in cats.

A significant moderate negative correlation (r = −0.66, 95% confidence interval: −0.76 to −0.53, *p* < 0.0001, *n* =100) was found between the abundance of *C. hiranonis* and the percentage of primary BAs (Figure 6). The median percentage of primary BAs was 14% (range: 1–70%) in the 78 samples from healthy cats, while the median percentage of primary BAs was 87% (range: 54–99%) in the 22 samples from cats receiving antibiotics.

## 4. Discussion

This study showed that the feline dysbiosis index in healthy adult cats remained within the reference interval (RI) over two months, despite subtle variation. The consistent positioning of the feline DI within the RI across 142 samples indicates temporal stability of the major bacterial taxa over two months. The PCA plot using the abundances of the targeted bacterial group as features shows that each cat’s microbiota remained consistent across multiple time points, compared to other cats. This finding aligns with a study in healthy cats using 16s rRNA gene sequencing [15] and studies in humans [9,14,41,42], in which the sampling date had a smaller effect size compared to the inter-individual variability. However, the tight clustering of samples from healthy cats was distinct from those with feline chronic enteropathy and cats that received antibiotics, suggesting that the inter-individual variation between healthy cats has a smaller effect when compared to the large shift in the microbiota in cats with dysbiosis.

The median coefficients of variation were below 6.2% in five out of seven targeted bacterial taxa (*Bacteroides*, *Bifidobacterium*, *C. hiranonis*, *Faecalibacterium*, and *Turicibacter*) for repeated testing for 17 healthy cats, indicating a relatively low degree of variability in the fecal abundances of the targeted bacteria in the healthy adult pet cats. Although higher coefficients of variation were observed in the abundance of *Streptococcus* and *E. coli*, these variations remained mostly within the RIs and were not associated with abnormal DI results. These findings were similar to a human study where *E. coli* showed high variation in fecal samples over time [42]. Additionally, the Human Microbiome Project discovered that while the taxonomic diversity and abundance of the microbiome vary among healthy adults, the functional composition of the microbiome is stable based on the sequencing data [43]. Another study in humans reported that the abundances of *Bifidobacterium*, *Faecalibacterium*, and most members of the phylum Bacteroidetes retained a high level of similarity over a decade, suggesting the existence and importance of the core community in humans [41] and dogs [44].

Despite numerous studies reporting statistical differences in various bacterial groups due to different factors, quantifying the effect size of these shifts remains challenging based on the sequencing results. When evaluating the impact of chronic inflammation or antibiotic-induced dysbiosis in the gastrointestinal tract, our results suggest that individual temporal variations have a limited impact on fecal microbiota in healthy populations. This aligns with observations in a previous study in cats [40], where factors like age, diet, and geographical location had a much smaller influence on fecal microbiota, compared to those with gastrointestinal diseases. From a clinical standpoint, the influence of individuality on the microbiota appears minimal when compared to the effects of disease, indicating the existence of a range of normality in bacterial taxon abundance.

The stable composition of intestinal microbiota is crucial to maintain gut health because the microbiota contributes to multiple physiological pathways. One of the most important pathways is BA metabolism [18,37,45]. Our study also found that the abundance of *C. hiranonis* was significantly associated with the composition of BAs in cats. A diverse spectrum of bacteria, such as *Lactobacillus* [46,47], *Bifidobacterium* [47], and *Bacteroides* [48], deconjugate the glycine- or taurine-conjugated primary BAs. Conversely, only a few species have been recognized as converting primary unconjugated BAs to secondary BAs. For example, *C. hiranonis* has been identified as a major contributor to this process in both dogs [49,50] and cats [51] and is included in the DI [40]. The association between the depletion of *C. hiranonis* and increased primary BAs has been repeatedly reported in dogs [30,49], cats with CE [31], and dogs receiving antibiotics [50]. Similarly, in this study, antibiotic exposure in cats possibly led to the loss of fecal abundance of *C. hiranonis* with a median of 0.1 log DNA, and an increased percentage of fecal primary BAs as high as 99% was observed. A significant correlation has been observed between the fecal abundance of *C. hiranonis* and the percentage of fecal primary BAs in this and other studies [49,50,51], suggesting *C. hiranonis* as the main BA convertor in cats. Notably, even in cats with relatively low abundance (below the lower limit of the RI, but above 3 log DNA), the conversion of BAs was present in all healthy cats [31]. Whether *C. hiranonis* is the only species that converts BA in cats requires further investigations. Interestingly, one cat that received antibiotics had loss of *C. hiranonis,* but the DI remained below 0, emphasizing the need for interpreting both the DI and the individual targeted bacterial groups, especially *C. hiranonis*, together. A study in dogs using 16s rRNA sequencing found that the distribution of the overall fecal microbiota (beta diversity) could be distinguished based on the absence or presence of *C. hiranonis* evaluated by qPCR [36].

In our study, cats receiving antibiotics showed the loss of fecal abundance of *C. hiranonis* and abnormal BA conversion that persisted for at least 2 months and up to 4 months (study duration), reflecting long-term perturbations in the gut microbiota. Their fecal microbiota was distinct from healthy cats, as shown in the clear separation on the PCA plot as well as the combination of the DI and the low abundance of *C. hiranonis*. Although these cats had no antibiotic-induced gastrointestinal signs, the clinical relevance of a long-term disturbance of the intestinal microbiota due to antibiotic administration warrants further research. Studies have shown that a stable microbiome is required for the colonization resistance of externally introduced bacteria and the expansion of opportunistic pathogens. This is exemplified by microbial-derived secondary BAs suppressing *Clostridium difficile* growth in dogs [52] and humans [33,53]. In kittens, oral antibiotics induced diarrhea and increased the susceptibility to enteropathogens [54]. Yet, kittens that did not receive antibiotics remained asymptomatic, emphasizing the importance of an intact microbiota for health. Collectively, studies from humans, dogs, and cats suggest that a consistently stable abundance of *C. hiranonis* might be linked to the normal composition of fecal microbiota and BAs.

Several limitations should be acknowledged. First, the owners were instructed to collect the samples within one day of defecation and store them in a freezer before taking them to the laboratory. Room temperature exposure might promote the growth of facultative bacteria [55]; however, a study showed that storage at room temperature for up to four days did not have a major effect on the feline fecal microbiota [56]. The abundance of *E. coli* was above the upper limit of the RI in just 1% of all samples in this study, indicating the minimal effect of exposure under a short-term room temperature environment. Second, the inclusion of only a single sample from cats with chronic enteropathy from a previous study does not allow for the evaluation of the temporal variability of fecal microbiota in cats with CE. Therefore, the samples from cats with CE from a previous study were used in this study only to add perspective to the magnitude of temporal variability observed in the PCA plots of healthy cats. Thirdly, though the owners were veterinarians or laboratory staff, diseases can be asymptomatic. The broad enrollment criteria might overlook subclinical changes, especially in geriatric cats. Nevertheless, all cats above 7 years of age in the current studies had a DI within the RI. A previous study also showed no significant correlation between age and DI in healthy adult pet cats [40]. Histopathological changes in the GI tracts of clinically healthy cats were reported [57]; however, the clinical relevance remains unclear.

## 5. Conclusions

In the absence of major perturbations, the feline dysbiosis index showed temporal stability over two months in healthy cats. All 17 healthy adult pet cats had the feline dysbiosis index within the reference interval throughout the collection period. The median coefficients of variation were less than 6.2% for five out of seven targeted bacterial groups in the 17 healthy cats. Despite relatively higher coefficients of variation for *E. coli* and *Streptococcus*, the abundances of *E. coli* and *Streptococcus* were above the upper reference limit in 1% and 0% of samples, respectively. This finding indicates the temporal stability of these predominant bacterial groups. Additionally, the microbiota of healthy cats clustered away from cats with CE and cats receiving antibiotics, suggesting the minimal magnitude of impact of the individual temporal variation on the fecal microbiota. Moreover, fecal bile acid profile was significantly associated with the abundance of *C. hiranonis*, indicating its role as a bile acid convertor in cats.

## Figures and Tables

**Table 1:** Descriptive data of the fecal abundances of the targeted bacterial groups of 142 samples from 17 healthy adult pet cats and the coefficient of variation of the bacterial abundances of each cat. Data expressed as the median (minimum–maximum).

Bacterial Group	Abundance (log DNA/g Feces) *n* = 142	Coefficient of Variation (%) *n* = 17	Number of Cats out of the Clinically Relevant Limit ^1^ *n* = 17
*Bacteroides*	7.6 (4.5–6.9)	5.5 (2.2–11.8)	0
*Bifidobacterium*	8.4 (4.2–6.9)	4.6 (1.8–16.4)	0
*C. hiranonis*	6.8 (3.1–5.5)	5.1 (0.7–14.8)	2
*Escherichia coli*	7.1 (0.9–2.8)	25.1 (5.9–61.2)	1
*Faecalibacterium*	7.4 (3.3–6.9)	4.7 (0.7–10.9)	1
*Streptococcus*	4.9 (0.7–2.8)	12.6 (2.1–27.4)	0
*Turicibacter*	8.0 (4.5–6.1)	6.2 (2–17.6)	0

^1^ Number of cats that had fecal abundances of *E. coli* and *Streptococcus* above the upper limit of their respective reference intervals or the abundances of the other targeted bacterial groups below the lower limit. This respective limit was based on their clinical relevance [40].

## Data Availability

Data is contained within the article and supplementary material.

## Supplementary Materials

The following are available online at https://www.mdpi.com/article/10.3390/vetsci11010031/s1, Excel Sheet-Collection Time Point and Excel Sheet-qPCR Condition.
